# Estimating dose-specific cell division and apoptosis rates from chemo-sensitivity experiments

**DOI:** 10.1038/s41598-018-21017-5

**Published:** 2018-02-09

**Authors:** Yiyi Liu, Forrest W. Crawford

**Affiliations:** 1 Department of Biostatistics, Yale School of Public Health, New Haven, USA; 2 Department of Ecology and Evolutionary Biology, Yale University, New Haven, USA; 3 Yale School of Management, New Haven, USA

## Abstract

*In-vitro* chemo-sensitivity experiments are an essential step in the early stages of cancer therapy development, but existing data analysis methods suffer from problems with fitting, do not permit assessment of uncertainty, and can give misleading estimates of cell growth inhibition. We present an approach (bdChemo) based on a mechanistic model of cell division and death that permits rigorous statistical analyses of chemo-sensitivity experiment data by simultaneous estimation of cell division and apoptosis rates as functions of dose, without making strong assumptions about the shape of the dose-response curve. We demonstrate the utility of this method using a large-scale NCI-DREAM challenge dataset. We developed an R package “bdChemo” implementing this method, available at https://github.com/YiyiLiu1/bdChemo.

## Introduction

In the early stages of cancer therapy development, potencies of candidate compounds are usually tested *in vitro* through chemo-sensitivity studies^1,2^. Researchers treat cultured tumor cells with different concentrations of compounds, and the numbers of cells remaining after a follow-up time *T* are recorded via fluorescent signal intensities that measure general metabolism levels or enzymatic activities^3^. Compounds that achieve desired tumor inhibition effects within dose ranges that are not considered clinically toxic are identified for further optimization and then tested with animal models and clinical trials^1^. Given the significant investment required to bring drug candidates to preclinical and clinical stages^4^, screening and selecting the most promising candidates from chemo-sensitivity studies is essential for drug development.

**Figure 1:**
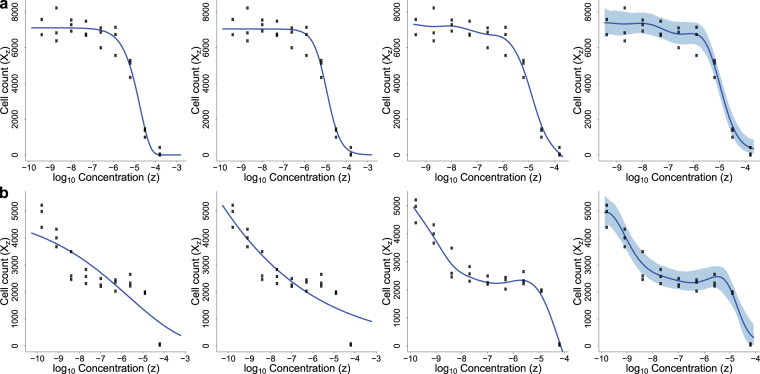
Dose-response modeling of cell line AU565 treated with compound 4-HC (**a**) and cell line SUM52PE treated with compound Everolimus (**b**), using Gompertz curve fitting (the first panel), logistic curve fitting (the second panel), grofit (the third panel) and bdChemo (the fourth panel). Solid circles and blue curves are data points and fitted curves, respectively. For bdChemo, curves and shades represent the posterior mean and the 95% credible interval of the Kendall Process mean, respectively.

Conventionally, the growth inhibition response of a cell line to a compound is modeled with a sigmoid curve. The most commonly used are the Gompertz^5,6^ and logistic curves^7,8^. Figure 1a (left and middle panels) illustrates a typical dataset (cell line AU565 treated with compound 4-HC^6^), with fitted Gompertz and logistic curves. Concentrations needed to achieve certain levels of inhibition effects, such as *GI*_50_ (growth of the cell population is inhibited by half), *TGI* (growth of the cell population is eliminated) and *LC*_50_ (half of the initial cell population is eliminated) are then estimated as assessments of the compound’s potency^9^.

However, this approach suffers from problems that may hinder its utility in chemo-sensitivity evaluation. First, it relies on a parametric form of the growth curve. Different compounds may affect cell growth in physiologically distinct ways makes it unreasonable to believe that all inhibition patters, which result from complex interactions between compounds and cells, could be modeled with the same parametric form (Fig. 1b, the first two panels). A newer nonparametric method called grofit^10^ provides a framework for fitting more flexible dose-response curves using spline smoothing. However, all these methods, by fitting a single growth curve, only deliver information about the combined effects of the compound on cell birth and death processes, while understanding these responses individually is critical to designing experiments that more elaborately investigate the compound’s mechanism of action^11^. Moreover, cancer therapy development often begins by designing compounds that target pathways either inhibiting cell division or inducing cell death separately^12^, so it would be helpful to separately discern these effects from early-stage chemo-sensitivity experiments. Finally, existing approaches consider only “point estimation” of *GI*_50_/*TGI*/*LC*_50_, as a summary of the compound inhibition effects without providing measures of uncertainty for these estimated quantities.

To overcome these limitations, we describe a new analysis approach (bdChemo) for chemo-sensitivity studies. This method specifies a mechanistic model of stochastic cell growth, fits semi-parametric dose-response curves without strong assumptions on their functional forms, and separately estimates dose-specific “birth” and “death” rates for the compound. For a given compound, we assume that a cell line’s per-cell birth and death rates, *λ* and *μ*, are time-homogenous and depend on the log_10_ concentration *z*, of the compound; a cell community with size *k* has aggregate population-level birth and death rates *kλ*(*z*) and *kμ*(*z*), respectively. Such a system is known as Kendall, or birth-death, process^13^ (BDP). When the BDP accurately characterizes of the dynamics of cellular response to the compound, the estimated rates of birth and death may have a mechanistic interpretation as “cell division” and “apoptosis” rates, respectively. To avoid unnecessary assumptions on the dose-dependent shapes of *λ*(*z*) and *μ*(*z*) and allow a flexible relationship between dose and response, we employ a semi-parametric Bayesian approach by assigning Gaussian process^14^ priors, which treats a regression function on a continuous domain as an infinite-dimensional random variable. The method assumes that any finite marginal distribution follows a multivariate Gaussian distribution without restrictions on the parametric form of the regression function. We estimate the dose-response relationships of the per-cell birth and death rates *λ*(*z*) and *μ*(*z*) as well as other model parameters using a Markov Chain Monte Carlo (MCMC) algorithm^15^. Uncertainty in estimates of birth and death rates is appropriately propagated into uncertainty in summary statistics like *GI*_50_, *TGI* and *LC*_50_.

We demonstrate the utility of this method on a large-scale chemosensitivity dataset from NCI-DREAM challenge containing cell population size measurements on 53 breast cancer cell lines treated by 28 compounds. In the original work, the authors fit a Gompertz curve^5^ for each experiment and calculated *GI*_50_ to quantify the sensitivity of the cell line to the compound. We apply bdChemo to the dataset and estimate the posterior mean as well as the 95% equal quantile credible intervals (CI) of chemosensitivity summary statistics *GI*_50_,*TGI* and *LC*_50_, and cell birth and death rates, *λ*(*z*) and *μ*(*z*), for each compound and cell line combination. We compare the results of the proposed method with those obtained by conventional Gompertz and logistic curve fitting approaches as well as grofit.

## Results

We analyze the data from NCI-DREAM drug sensitivity prediction challenge^6^ to demonstrate the utility of estimates produced by the proposed method. This dataset contains dose-response measurements of 28 compounds on 53 breast cancer cell lines. Cells were treated with 9 doses of each compound in triplicate and cell counts at 72 h post treatment were measured using the Cell Titer Glo assay. In the original work, the authors fit a Gompertz curve^5^ for each experiment (a cell line treated by a compound) and calculated *GI*_50_ (a point estimate without uncertainty evaluation) to quantify the sensitivity of the cell line to the compound. We summarize the posterior mean and 95% equal quantile credible intervals (CI) of *λ*(*z*), *μ*(*z*), *GI*_50_,*TGI* and *LC*_50_ returned by bdChemo in Supplementary Table S1 and Figure S1.

### bdChemo fits dose-response curve flexibly

Conventional Gompertz and logistic curve fitting approaches rely on specified parametric forms of the dose-response curve. These parametric forms may not be flexible enough to describe the observed dose-response data due to the complex interactions between compounds and cell lines. Compared to the restricted parametric curve fitting approaches, bdChemo does not put strong assumptions on the functional forms of the dose-response curve, and hence provides a flexible and data-driven approach to study the effect of a compound on a cell line.

Figure 1a and b depict two examples of different growth patterns modeled by the four approaches. For compound 4-HC working on cell line AU565 (Fig. 1a), the growth curve follows a sigmoid shape. In such case, all four approaches produced good fits. However, for compound Everolimus working on cell line SUM52PE (Fig. 1b), where there is a plateau around the waist of the growth curve, the two sigmoid curve fitting approaches (the first two panels) could not capture this trend, while grofit (the third panel) and bdChemo (the fourth panel) generated curves more concordant with the data.

### bdChemo provides separate estimates of cell birth and death rates

**Figure 2:**
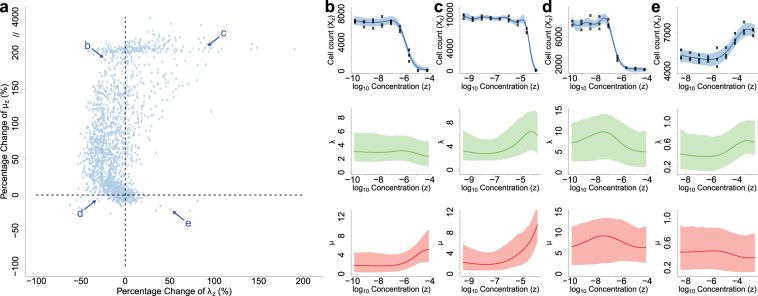
(**a**) Percentage change between the posterior mean estimates of death rates (*μ*(*z*)) at the largest and the smallest tested concentrations v.s percentage change between posterior means of birth rates (*λ*(*z*)). (**b–e**) Posterior estimations of Kendall Process means (dose-response curves, upper panel), birth rates (middle panel) and death rates (lower panel) of cell line HCC1954 treated with compound IKK 16 (**b**), cell line MCF7 treated with compound 4-HC (**c**), cell line HCC1428 treated with compound MG-132 (**d**) and cell line HCC202 treated with compound PS-1145 (**e**). Curves and shades represent the posterior mean and the 95% credible interval of the corresponding variables, respectively.

Together, cell division and apoptosis, as functions of compound dose, determine the dynamics of cell line response. For these distinct cellular processes, model-based estimation of compounds’ effects on cell birth and death rates can serve as a screening tool for candidate compounds and provide guidance for hypothesis generation and experiment design to study compounds’ cellular mechanisms of action. We depict the percentage changes between the posterior means of birth/death rates at the largest and the smallest tested concentrations in Figure 2a (all compounds) and Figure S2 (individual compound). The overall growth, birth and death curves of four examples are included in Figure 2b–e as further illustrations. When treated by a compound, most cell lines in this study show decreased birth and increased death rates under higher concentrations (top left part of Figure 2a; an example in Figure 2b), while a few exhibit only decreased birth rates or increased death rates alone (top right and bottom left parts in Figure 2a; examples in Figure 2c,d). Not all compounds in this study appear to inhibit cell growth, resulting in a few other points with large birth rate increment or death rate decrement effect (bottom right part in Figure 2a; an example in Figure 2e).

**Figure 3:**
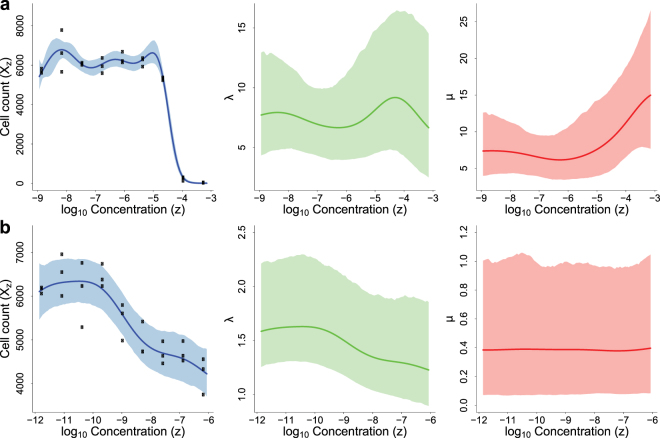
Posterior estimations of Kendall Process means (dose-response curves, left panel), birth rates (middle panel) and death rates (right panel) of cell line BT474 treated with compound Imatinib (**a**) and cell line HCC1806 treated with compound Cetuximab (**b**). Curves and shades represent the posterior mean and the 95% credible interval of the corresponding variables, respectively.

These scenarios may suggest different underlying mechanisms of action. For example, in several other cancers, Imatinib has been reported to kill tumor cells by decreasing the activity of tyrosine kinase enzymes^16^, whereas Cetuximab was known to hinder uncontrolled tumor cell division as an EGFR inhibitor^17^; here we observe similar effects on breast cancer cell lines: for the compound Imatinib and cell line BT474 (Figure 3a), birth rate *λ*(*z*) is relatively stable with respect to dose *z* in the tested range, while death rate *μ*(*z*) first stays steady but then increases rapidly when dose *z* becomes large; in contrast, for Cetuximab working on cell line HCC1806 (Figure 3b), birth rate *λ*(*z*) decreases while death rate *μ*(*z*) stays stable. Therefore, we may hypothesize that compound Imatinib mainly works by inducing cell apoptosis on BT474 while compound Cetuximab is more likely to target on blocking cell division on HCC1806, and design experiments to investigate the effects of these compounds on cell apoptosis and division-related pathways, respectively, to better understand their mechanisms on these cell lines. Similar cell death induction and birth inhibition effects of Imatinib and Cetuximab are also observed on most other cell lines in this dataset (Figure S2). In addition, some other compounds have consistent patterns across cell lines. For example, Mebendazole, TCS PIM-11, QNZ and MG-132 demonstrate both birth inhibition and death induction effects on most cell lines. Other compounds like 4-HC, Doxorubicin, Olomoucine II, Valproate, Baicalein, Methylglyoxal and IKK 16 increase cell death rates on most cell lines, but their effects on cell birth rates differ by cell line.

### bdChemo considers uncertainty in chemosensitivity evaluations

**Figure 4:**
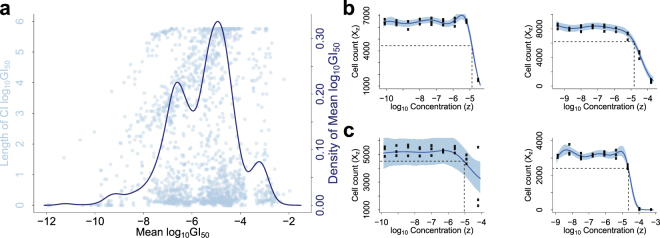
(**a**) Length of the credible interval of log_10_*GI*_50_v.s. mean of log_10_*GI*_50_; distribution of mean log_10_*GI*_50_. (**b,c**) Dose-response modeling of cell line 21NT treated with compounds Nelfinavir and 4-HC (**b**), and cell line MDAMB415 treated with compounds Disulfiram and Imatinib (**c**). Curves and shades in (**b,c**) represent the posterior means and the 95% credible intervals of the Kendall Process means, respectively. Dashed lines are the log_10_*GI*_50_’s calculated from the mean estimations of Kendall Process means.

Point estimates of compound potency summary statistics, such as *GI*_50_, on different experiments can be similar even if the growth curves have distinct patterns. In Figure 4a, we plot the lengths of the 95% credible intervals (CI) against the means for log_10_*GI*_50_ estimated on all experiments from the NCI-DREAM data. There are many cases where means are comparable but lengths of credible intervals differ greatly. For example, on cell line 21NT, compounds Nelfinavir and 4-HC have *GI*_50_ values 1.33 × 10^−5^*M* and 1.29 × 10^−5^*M* respectively, which are close (especially in log_10_ scale the difference is negligible), yet Nelfinavir has a 95% CI [1.17 × 10^−5^,1.53 × 10^−5^]*M*, much narrower than that of 4-HC, [8.87 × 10^−6^,1.74 × 10^−5^]*M*. These differences result from the distinct patterns of their growth curves: compared to the sharp drop of the growth curve under treatment of Nelfinavir (Figure 4b left panel), the growth curve under treatment of 4-HC declines much more slowly as compound concentration increases (Figure 4b right panel), resulting in greater uncertainty about the location of *GI*_50_.

Comparing point estimates of a summary statistic for different compounds can lead to conclusions about their relative potency that lack statistical validity. For instance, on cell line MDAMB415, compounds Disulfiram and Imatinib have *GI*_50_ values 6.55 × 10^−6^*M* and 2.28 × 10^−5^*M*, respectively (Figure 4c), based on which, a conclusion may be drawn that MDAMB415 cells are more sensitive to Disulfiram. However, as the *GI*_50_ CI of Disulfiram is [1.36 × 10^−10^,2.67 × 10^−5^]*M*, covering that of Imatinib, [2.00 × 10^−5^,2.62 × 10^−5^]*M*, there is no evidence supporting the statistical significance of such difference. In practice, summary concentrations are often computed to quickly compare a large number of compounds. However, rigorous statistical hypothesis testing for differences in cell line responses requires taking into account uncertainties in summary statistics used for comparison.

## Discussion

Statistically rigorous analysis of chemosensitivity experiment data is of great importance in cancer therapy development. The major assumptions of the proposed model, bdChemo, based on the birth-death process^13^ (BDP), are that the per-cell birth and death rates of a cell line under compound treatment are time-homogenous functions of the compound concentration, and the population-level birth and death rates are products of the cell community size and the per-cell birth and death rates, respectively. Unlike standard analysis methods that rely heavily on specific functional forms of the dose-response curve, bdChemo employs a semi-parametric Bayesian approach in function estimations to avoid restrictive assumptions. Although other nonparametric methods have been proposed for dose-response curve fitting, bdChemo provides biologically motivated estimates of dose-dependence in cell birth and death rates separately, in addition to estimating the combined effects of the compound on cell birth and death processes, delivering richer information that may guide subsequent experimental work. The method takes uncertainty into account when providing chemosensitivity summary statistics, such as *GI*_50_, *TGI* and *LC*_50_, to facilitate sound comparisons of compounds’ effects in tumor cell growth inhibition.

We applied bdChemo, as well as two conventional sigmoid-curve fitting approaches (logistic and Gompertz) and R package grofit^10^, to NCI-DREAM drug sensitivity prediction challenge^6^ data, where dose-response measurements of 28 compounds on 53 breast cancer cell lines were provided. The results show that when the dose-response curve does follow a sigmoid pattern, bdChemo produces estimates similar to conventional methods; but when the dose-response curve deviates from a sigmoidal shape, bdChemo and nonparametric spline smoothing can better capture growth inhibition dynamics. We observe different patterns of cell birth inhibition and death induction effects for difference cell line/compound combinations; while some compound-cell line combinations have dose-response curves that look similar, the underlying mechanism of action may be different. Separate estimates of cell birth and death rate estimations provided by bdChemo, hence, might be utilized in hypothesis generation and experimental design. In addition, even when the point estimates of *GI*_50_ are similar, credible intervals sometimes differ substantially.

While bdChemo can fit curves flexibly and provides mechanistic inferences about the dose-dependent respond of cell birth and death rates, the approach is subject to limitations. First, the Kendall process framework assumes that cells undergo birth and death independently, with the same per-cell rates; when there are *k* cells, the population-level birth and death rates are *kλ*(*z*) and *kμ*(*z*) respectively. The model does not accommodate population-level effects, which could result in more complicated nonlinear rates *λ*(*k*,*z*) and *μ*(*k*,*z*). More sophisticated models for population effects of cell response may be warranted when biologically motivated. Second, we analyzed each experiment (compound-cell line combination) independently. Jointly modeling inhibition responses by compound and cell line could exploit information sharing across experiments, resulting in greater statistical precision in estimates, especially when the number of doses is small.

Finally, we point out potential issues that may arise when applying bdChemo in empirical data analysis. We observed some large credible intervals of *λ* and *μ* in our analysis, which is mainly caused by the small number of experimental replicates (three at each dose). Since the mean value of the cell numbers only contains information regarding the combined effects of the birth and death processes, the separate identifiability of the birth and death rates mainly comes from the variance of cell numbers at different doses. Therefore, a larger number of replicates is desirable for increased accuracy in estimates of *λ* and *μ*. Additionally, we observed a few outliers in which *λ* and *μ* are estimated to be much larger than elsewhere (Tables S2 and S3). These estimates are driven by large cell count variations in these experiments. For a comprehensive evaluation of our method’s performance on datasets of varying qualities, we analyzed all NCI-DREAM experiments here. However, in practice, some quality control procedures in data preprocessing, as typically conducted in conventional chemosensitivity analysis^18^, might also be necessary.

## Methods

### Conventional approaches

Conventionally a cell line’s response to a compound is modeled with a sigmoid curve. The most commonly used include Gompertz curve^5,6^

1 $$\begin{array}{c}g(z)={\phi }_{0}+{\phi }_{1}\exp \,(-\exp ({\phi }_{2}+{\phi }_{3}z))\end{array},$$

and logistic curve^7,8^

2 $$\begin{array}{c}g(z)={\psi }_{0}+\frac{{\psi }_{1}}{1+\exp (\frac{z-{\psi }_{2}}{{\psi }_{3}})},\,\end{array}$$

where *g*(*z*) is the final cell count at log_10_ concentration, *z* (usually with unit log_10_ M).

### bdChemo

#### Model

We use birth-death process (BDP) to model cell growth in the experiment. In a general BDP^19^, where *N*(*t*) stands for the number of particles at time *t*, given *N*(*t*) = *k*(*k* ≥ 1), the birth rate

3 $$\begin{array}{c}{\lambda }_{k}=\mathop{\mathrm{lim}}\limits_{dt\to 0}\frac{{\rm{\Pr }}\,(N(t+dt)=k+1|N(t)=k)}{dt}\end{array}$$

and the death rate

4 $$\begin{array}{c}{\mu }_{k}=\mathop{\mathrm{lim}}\limits_{dt\to 0}\frac{{\rm{\Pr }}\,(N(t+dt)=k-1|N(t)=k)}{dt}\end{array}$$

are time-homogeneous but dependent on the number of particles *k*. A simple linear BDP known as Kendall Process assumes *λ*_*k*_ = *λk* and *μ*_*k*_ = *μk* (we refer *λ* and *μ* as per cell birth and death rates, respectively)^13^. For Kendall Process, the transition probability is^20^

5 $$\begin{array}{rcl}P{}_{ab}(t) & = & {\rm{\Pr }}(N(t)=b|N(0)=a)\\  & = & \sum _{j=0}^{{\rm{\min }}(a,b)}(\begin{array}{c}a\\ j\end{array})(\begin{array}{c}a+b-j-1\\ a-1\end{array}){\alpha }^{a-j}{\beta }^{b-j}{(1-\alpha -\beta )}^{j},\end{array}$$

where

6 $$\begin{array}{c}\alpha (t)=\frac{\mu ({e}^{(\lambda -\mu )t}-1)}{\lambda {e}^{(\lambda -\mu )t}-\mu }\end{array}$$

and

7 $$\begin{array}{c}\beta (t)=\frac{\lambda ({e}^{(\lambda -\mu )t}-1)}{\lambda {e}^{(\lambda -\mu )t}-\mu }\end{array}$$

For a fixed *t*, *N*(*t*) is a random variable whose distribution is fully specified by *P*_*ab*_(*t*). The mean and variance of *N*(*t*) given *N*(0) = *n*_0_ are^19,21^

8 $$\begin{array}{c}{m}_{t}={n}_{0}\exp ((\lambda -\mu )t)\end{array}$$

and

9 $$\begin{array}{c}{v}_{t}={n}_{0}\frac{\lambda +\mu }{\lambda -\mu }\exp \,((\lambda -\mu )t)[\exp \,((\lambda -\mu )t)-1],\end{array}$$

respectively.

As indicated above, to calculate *P*_*ab*_(*t*) involves computing a large number of combinatorial numbers, making it computationally infeasible in real applications^22^. Therefore, we use normal distribution with matched mean (*m*_*t*_) and variance (*v*_*t*_) as an approximation to reduce the computational cost. This approximation is accurate when initial cell counts are large, as they generally are in chemosensitivity experiments (see the Supplementary Note for a technical justification). Since in one chemo-sensitivity study, treatment duration *t* is usually fixed and same for all experiments (compounds), we omit the notation *t* for simplicity and interpret the new *λ* and *μ* as per cell birth and death rates for the entire experiment duration.

For a given compound working on a given cell line, we assume *λ* and *μ* are functions of log_10_ concentration, *z*, of the compound, so the mean and variance of cell counts are

10 $$\begin{array}{c}m({n}_{0},z)={n}_{0}\exp \,(\lambda (z)-\mu (z)),\end{array}$$

and

11 $$\begin{array}{c}v({n}_{0},z)={n}_{0}\frac{\lambda (z)+\mu (z)}{\lambda (z)-\mu (z)}\exp (\lambda (z)-\mu (z))[\exp \,(\lambda (z)-\mu (z))-1],\end{array}$$

respectively.

Note, although the mean as a function of *λ* and *μ* is only affected by their difference, the variance has a term *λ* + *μ*. Therefore, mean and variance of cell counts together provides information to identify *λ* and *μ* separately. An interesting example is illustrated by the difference between quiescence (*λ* ≈ 0 and *μ* ≈ 0) and matched birth and death rates (*λ* ≈ *μ*). In both cases, we would expect the mean cell count to be unchanged, *m*(*n*_0_, *z*) ≈ *n*_0_. However, if both *λ* and *μ* are close to 0, the variance *v*(*n*_0_,*z*) would also be very small; if the rates are large and nearly equal, *λ* ≈ *μ* ≫ 0, the variance is expected to be much larger.

Our model also takes experimental errors into account by assuming that the measured cell counts *X*_*z*_ = *N*_*z*_ + *∈*_*z*_ and *∈*_*z*_ ∼ *N*(*θ*,*σ*^2^), where *θ* and *σ*^2^ are the mean and variance of background noises, respectively.

Therefore, the likelihood of observing data *D* = {(*n*_01_, *z*_1_, *x*_1_), (*n*_02_, *z*_2_, *x*_2_), …, (*n*_0*n*_, *z*_*n*_, *x*_*n*_), *e*_1_, …, *e*_*q*_} (*n*_0*i*_’s are the initial cell population sizes, (*z*_*i*_, *x*_*i*_)’s are compound concentrations and corresponding cell count measurements at follow-up, and *e*_*i*_’ s are independent background noise measurements) is

12 $$\begin{array}{c}L(D|\lambda ,\mu ,{\sigma }^{2})=\prod _{i=1}^{n}{f}_{N}({x}_{i}|m({n}_{0i},{z}_{i})+\theta ,v({n}_{0i},{z}_{i})+{\sigma }^{2})\prod _{i=1}^{q}{f}_{N}({e}_{i}|\theta ,{\sigma }^{2}),\end{array}$$

where *f*_*N*_ is the density function of Gaussian distribution.

Note that we do not require the initial cell population sizes *n*_0*i*_ to be the same across different compound concentrations, but we treat them as known quantities here. In reality, initial cell population sizes may be uncertain in some experiments. However, with only one number provided in most experiment data, it is difficult to assign a distribution to the initial cell count. Moreover, uncertainty in the initial count mainly comes from variation in cell density and the amount of cell solution injected into each well, both of which, under appropriate experimental operation, could be controlled at a relatively low level. Although we do not model its randomness directly, the term *σ*^2^ models the variance from background noise can be interpreted to reflect variation in initial cell counts to some extent. For example, if two datasets have all other conditions similar, whereas one has a much larger estimate of *σ*^2^, we might suspect that the large *σ*^2^ is induced by a poorly controlled initial cell seeding. If future experiments provide more data to quantify its fluctuations and suggest the necessity of treating it as a random variable, we may add another layer of randomness to *n*_0_ under this Bayesian hierarchical model framework.

To avoid unnecessary assumptions on *λ*(*z*) and *μ*(*z*) and allow more flexible estimations, we employ a semi-parametric Bayesian approach by assigning Gaussian process^14^ priors to *ϕ*_*λ*_(*z*) = log(*λ*(*z*)) and *ϕ*_*μ*_(*z*) = log(*μ*(*z*)), i.e.

13 $$\begin{array}{c}{\varphi }_{\lambda }(z)\sim GP({\alpha }_{\lambda },{K}_{\lambda }),\end{array}$$

14 $$\begin{array}{c}{\varphi }_{\mu }(z)\sim GP({\alpha }_{\mu },{K}_{\mu }),\end{array}$$

and

15 $$\begin{array}{c}{K}_{\lambda }(z,z^{\prime} )={\tau }_{\lambda }^{2}\exp (-\frac{{(z-z^{\prime} )}^{2}}{{l}_{\lambda }^{2}}),\end{array}$$

16 $$\begin{array}{c}{K}_{\mu }(z,z^{\prime} )={\tau }_{\mu }^{2}\exp (-\frac{{(z-z^{\prime} )}^{2}}{{l}_{\mu }^{2}}).\end{array}$$

Note, unlike conventional approaches, under this Gaussian process framework, we impose no shape constraints including monotonicity on the curves.

Hyperparameters ${\alpha }_{\lambda },{\alpha }_{\mu },{\tau }_{\lambda }^{2},{\tau }_{\mu }^{2},{l}_{\lambda }^{2},{l}_{\mu }^{2},\theta$ and *σ*^2^are assigned priors

17 $$\begin{array}{c}{\alpha }_{\lambda }\sim N(0,s{a}_{\lambda }^{2}),\,\end{array}$$

18 $$\begin{array}{c}{\alpha }_{\mu }\sim N(0,s{a}_{\mu }^{2}),\end{array}$$

19 $$\begin{array}{c}{\tau }_{\lambda }^{2}\sim Inverse-Gamma({a}_{1\lambda },{b}_{1\lambda }),\,\end{array}$$

20 $$\begin{array}{c}{\tau }_{\mu }^{2}\sim Inverse-Gamma({a}_{1\mu },{b}_{1\mu }),\end{array}$$

21 $$\begin{array}{c}{l}_{\lambda }^{2}\sim Gamma({a}_{2\lambda },{b}_{2\lambda }),\end{array}$$

22 $$\begin{array}{c}{l}_{\mu }^{2}\sim Gamma({a}_{2\mu },{b}_{2\mu }),\end{array}$$

23 $$\begin{array}{c}\theta \sim Unif(-\infty ,\infty ),\,i.e.\,p(\theta )\propto 1\end{array}$$

and

24 $$\begin{array}{c}{\sigma }^{2}\sim log-Unif(0,1),\,i.e.\,p({\sigma }^{2})\propto \frac{1}{{\sigma }^{2}}.\end{array}\,$$

#### Fitting algorithm

We utilize Gibbs sampler embedded with Metropolis updating^15^ to draw posterior samples for parameters ${\varphi }_{\lambda },{\varphi }_{\mu },{\alpha }_{\lambda },{\alpha }_{\mu },{\tau }_{\lambda }^{2},{\tau }_{\mu }^{2},{l}_{\lambda }^{2},{l}_{\mu }^{2},\theta$ and *σ*^2^ (see Supplementary Note for details).

### Data availability

The dataset analyzed during the current study is available at http://www.nature.com/nbt/journal/v32/n12/full/nbt.2877.html. The code used to perform the analysis is available as an R package “bdChemo” at https://github.com/YiyiLiu1/bdChemo.

## Electronic supplementary material

Supplementary information

Table S1
