# Supplementary material for: Estimating dose-specific cell division and apoptosis rates from chemo-sensitivity experiments
Source: Sci Rep. 2018 Feb 9;8:2705. doi: 10.1038/s41598-018-21017-5 (PMC5807362; doi:10.1038/s41598-018-21017-5)
Supplement: Supplementary file 1 — Supplementary information [file 41598_2018_21017_MOESM1_ESM.pdf]

Estimating dose-specific cell division and apoptosis rates from chemo-sensitivity experiments

Yiyi Liu and Forrest W. Crawford

Supplementary Information

Justification of Normal estimation of Kendall Process transition probabilities

Kendall Process with initial population size $N_0 = n_0$ can be interpreted as the sum of n_0 independent Kendall Processes with same parameters and initial population size 1[2], i.e., $X_t = \sum_i^{n_0} Z_{it}$, where $Z_{it}, i = 1, 2, \cdots, n_0$ are from i.i.d. Kendall Processes with Z_{i0}=1. Denote

$$\mathbb{E}(Z_{it}) = \gamma_t, \tag{1}$$

$$var(Z_{it}) = \delta_t^2. \tag{2}$$

We have

$$m_t = n_0 \gamma_t, \tag{3}$$

$$v_t = n_0 \delta_t^2. \tag{4}$$

Using central limit theorem,

$$\frac{\left(\frac{X_t}{n_0} - \gamma_t\right)}{\frac{\delta_t}{\sqrt{x_0}}} \xrightarrow{D} N(0,1), as\ n_0 \to +\infty \tag{5}$$

i.e.

$$\frac{X_t - m_t}{\sqrt{v_t}} \xrightarrow{D} N(0,1). \tag{6}$$

Model fitting algorithm

We iteratively sample:

- $\phi_\lambda \mid \phi_\mu, \alpha_\lambda, \alpha_\mu, l_\lambda^2, l_\mu^2, \tau_\lambda^2, \tau_\mu^2, \theta, \sigma^2, D$:

$$\begin{aligned} &f(\phi_\lambda \mid \phi_\mu, \alpha_\lambda, \alpha_\mu, l_\lambda^2, l_\mu^2, \tau_\lambda^2, \tau_\mu^2, \theta, \sigma^2, D) \\ &= f(\phi_\lambda \mid \phi_\mu, \alpha_\lambda, l_\lambda^2, \tau_\lambda^2, \theta, \sigma^2, D) \\ &\propto L(D \mid \lambda, \mu, \theta, \sigma^2) \cdot f_N(\phi_\lambda \mid \alpha_\lambda \cdot \mathbf{1}, K_\lambda). \end{aligned} \quad (7)$$

  - Sample $\phi_{\lambda 1}$ from $N(\phi_{\lambda 0}, \Sigma_\lambda)$, where $\phi_{\lambda 0}$ is ϕ_λ from previous iteration.
  - Accept $\phi_{\lambda 1}$ with probability

$$r = \min\left\{1, \frac{f(\phi_{\lambda 1} \mid \phi_\mu, \alpha_\lambda, l_\lambda^2, \tau_\lambda^2, \theta, \sigma^2, D)}{f(\phi_{\lambda 0} \mid \phi_\mu, \alpha_\lambda, l_\lambda^2, \tau_\lambda^2, \theta, \sigma^2, D)}\right\}. \quad (8)$$

- $\phi_\mu \mid \phi_\lambda, \alpha_\lambda, \alpha_\mu, l_\lambda^2, l_\mu^2, \tau_\lambda^2, \tau_\mu^2, \theta, \sigma^2, D$:

$$\begin{aligned} &f(\phi_\mu \mid \phi_\lambda, \alpha_\lambda, \alpha_\mu, l_\lambda^2, l_\mu^2, \tau_\lambda^2, \tau_\mu^2, \theta, \sigma^2, D) \\ &= f(\phi_\mu \mid \phi_\lambda, \alpha_\mu, l_\mu^2, \tau_\mu^2, \theta, \sigma^2, D) \\ &\propto L(D \mid \lambda, \mu, \theta, \sigma^2) \cdot f_N\big(\phi_\mu \,\big|\, \alpha_\mu \cdot \mathbf{1}, K_\mu\big). \end{aligned} \quad (9)$$

  - Sample $\phi_{\mu 1}$ from $N\big(\phi_{\mu 0}, \Sigma_\mu\big)$, where $\phi_{\mu 0}$ is ϕ_μ from previous iteration.
  - Accept $\phi_{\mu 1}$ with probability

$$r = \min\left\{1, \frac{f(\phi_{\mu 1} \mid \phi_\lambda, \alpha_\mu, l_\mu^2, \tau_\mu^2, \theta, \sigma^2, D)}{f(\phi_{\mu 0} \mid \phi_\lambda, \alpha_\mu, l_\mu^2, \tau_\mu^2, \theta, \sigma^2, D)}\right\}. \quad (10)$$

- $\sigma^2 \mid \phi_\lambda, \phi_\mu, \alpha_\lambda, \alpha_\mu, l_\lambda^2, l_\mu^2, \tau_\lambda^2, \tau_\mu^2, \theta, D$

$$\begin{aligned} &f(\sigma^2 \mid \phi_\lambda, \phi_\mu, \alpha_\lambda, \alpha_\mu, l_\lambda^2, l_\mu^2, \tau_\lambda^2, \tau_\mu^2, \theta, D) \\ &= f(\sigma^2 \mid \phi_\lambda, \phi_\mu, \theta, D) \\ &\propto L(D \mid \lambda, \mu, \theta, \sigma^2) \cdot \frac{1}{\sigma^2}. \end{aligned} \quad (11)$$

  - Sample σ_1^2 from $N(\sigma_0^2, v^2)$, where σ_0^2 is σ^2 from previous iteration.
  - If $\sigma_1^2 < 0$, stay unchanged.
  - Otherwise, accept σ_1^2 with probability

$$r = \min\left\{1, \frac{f(\sigma_1^2 \mid \phi_\lambda, \phi_\mu, D)}{f(\sigma_0^2 \mid \phi_\lambda, \phi_\mu, D)}\right\}. \quad (12)$$

- $\theta \mid \phi_\lambda, \phi_\mu, \alpha_\lambda, \alpha_\mu, l_\lambda^2, l_\mu^2, \tau_\lambda^2, \tau_\mu^2, \sigma^2, D$

$$\begin{aligned} &f(\theta \mid \phi_\lambda, \phi_\mu, \alpha_\lambda, \alpha_\mu, l_\lambda^2, l_\mu^2, \tau_\lambda^2, \tau_\mu^2, \sigma^2, D) \\ &= f(\theta \mid \phi_\lambda, \phi_\mu, \sigma^2, D) \\ &\propto L(D \mid \lambda, \mu, \theta, \sigma^2) \cdot 1 \\ &\propto f_N\left(\theta \,\middle|\, \frac{\frac{\sum_{i=1}^q e_i}{\sigma^2} + \sum_{i=1}^n \frac{x_i - m_i}{\sigma^2 + v_i}}{\frac{q}{\sigma^2} + \sum_{i=1}^n \frac{1}{\sigma^2 + v_i}}, \frac{1}{\frac{q}{\sigma^2} + \sum_{i=1}^n \frac{1}{\sigma^2 + v_i}}\right). \end{aligned} \quad (13)$$

  - Update θ with a sample from

$$N\left(\frac{\frac{\sum_{i=1}^q e_i}{\sigma^2} + \sum_{i=1}^n \frac{x_i - m_i}{\sigma^2 + v_i}}{\frac{q}{\sigma^2} + \sum_{i=1}^n \frac{1}{\sigma^2 + v_i}}, \frac{1}{\frac{q}{\sigma^2} + \sum_{i=1}^n \frac{1}{\sigma^2 + v_i}}\right). \quad (14)$$

- $\alpha_\lambda \mid \phi_\lambda, \phi_\mu, \alpha_\mu, l_\lambda^2, l_\mu^2, \tau_\lambda^2, \tau_\mu^2, \theta, \sigma^2, D$

$$\begin{aligned} & f(\alpha_\lambda \mid \phi_\lambda, \phi_\mu, \alpha_\mu, l_\lambda^2, l_\mu^2, \tau_\lambda^2, \tau_\mu^2, \theta, \sigma^2, D) \\ & = f(\alpha_\lambda \mid \phi_\lambda, l_\lambda^2, \tau_\lambda^2) \\ & \propto f_N(\phi_\lambda \mid \alpha_\lambda \cdot \mathbf{1}, K_\lambda) \cdot f_N(\alpha_\lambda \mid 0, sa_\lambda^2) \\ & \propto f_N(\alpha_\lambda \mid \frac{\mathbf{1}^T K_\lambda^{-1} \phi_\lambda}{\mathbf{1}^T K_\lambda^{-1} \mathbf{1} + \frac{1}{sa_\lambda^2}}, \frac{1}{\mathbf{1}^T K_\lambda^{-1} \mathbf{1} + \frac{1}{sa_\lambda^2}}). \end{aligned} \tag{15}$$

  - Update α_λ with a sample from

$$N\left(\frac{\mathbf{1}^T K_\lambda^{-1} \phi_\lambda}{\mathbf{1}^T K_\lambda^{-1} \mathbf{1} + \frac{1}{sa_\lambda^2}}, \frac{1}{\mathbf{1}^T K_\lambda^{-1} \mathbf{1} + \frac{1}{sa_\lambda^2}}\right). \tag{16}$$

- $\alpha_\mu \mid \phi_\lambda, \phi_\mu, \alpha_\lambda, l_\lambda^2, l_\lambda^2, \tau_\lambda^2, \tau_\mu^2, \theta, \sigma^2, D$

$$\begin{aligned} & f\left(\alpha_\mu \mid \phi_\lambda, \phi_\mu, \alpha_\lambda, l_\lambda^2, l_\mu^2, \tau_\lambda^2, \tau_\mu^2, \theta, \sigma^2, D\right) \\ & = f\left(\alpha_\mu \mid \phi_\mu, l_\mu^2, \tau_\mu^2\right) \\ & \propto f_N\left(\phi_\mu \mid \alpha_\mu \cdot \mathbf{1}, K_\mu\right) \cdot f_N(\alpha_\mu \mid 0, sa_\mu^2) \\ & \propto f_N(\alpha_\mu \mid \frac{\mathbf{1}^T K_\mu^{-1} \phi_\mu}{\mathbf{1}^T K_\mu^{-1} \mathbf{1} + \frac{1}{sa_\mu^2}}, \frac{1}{\mathbf{1}^T K_\mu^{-1} \mathbf{1} + \frac{1}{sa_\mu^2}}). \end{aligned} \tag{17}$$

  - Update α_μ with a sample from

$$N\left(\frac{\mathbf{1}^T K_\mu^{-1} \phi_\mu}{\mathbf{1}^T K_\mu^{-1} \mathbf{1} + \frac{1}{sa_\mu^2}}, \frac{1}{\mathbf{1}^T K_\mu^{-1} \mathbf{1} + \frac{1}{sa_\mu^2}}\right). \tag{18}$$

- $\tau_\lambda^2 \mid \phi_\lambda, \phi_\mu, \alpha_\lambda, \alpha_\mu, l_\lambda^2, l_\mu^2, \tau_\mu^2, \theta, \sigma^2, D$

$$\begin{aligned} & f\left(\tau_\lambda^2 \mid \phi_\lambda, \phi_\mu, \alpha_\lambda, \alpha_\mu, l_\lambda^2, l_\mu^2, \tau_\mu^2, \theta, \sigma^2, D\right) \\ & = f\left(\tau_\lambda^2 \mid \phi_\lambda, \alpha_\lambda, l_\lambda^2\right) \\ & \propto f_N(\phi_\lambda \mid \alpha_\lambda \cdot \mathbf{1}, K_\lambda) \cdot f_{IG}(\tau_\lambda^2 \mid a_{1\lambda}, b_{1\lambda}) \\ & \propto f_{IG}\left(\tau_\lambda^2 \mid \frac{d}{2} + a_{1\lambda}, \frac{(\phi_\lambda - \alpha_\lambda \cdot \mathbf{1})^T G_\lambda^{-1} (\phi_\lambda - \alpha_\lambda \cdot \mathbf{1})}{2} + b_{1\lambda}\right), \end{aligned} \tag{19}$$

where f_{IG} is the density function of inverse-Gamma distribution, d is the number of distinct z_i's ($i = 1,2, \cdots, n$) and $G_\lambda = K_\lambda / \tau_\lambda^2$.

  - Update τ_λ^2 with a sample from

$$IG\left(\frac{d}{2} + a_{1\lambda}, \frac{(\phi_\lambda - \alpha_\lambda \cdot \mathbf{1})^T G_\lambda^{-1} (\phi_\lambda - \alpha_\lambda \cdot \mathbf{1})}{2} + b_{1\lambda}\right). \tag{20}$$

- $\tau_\mu^2 \mid \phi_\lambda, \phi_\mu, \alpha_\lambda, \alpha_\mu, l_\lambda^2, l_\mu^2, \tau_\lambda^2, \theta, \sigma^2, D$

$$\begin{aligned} & f\left(\tau_\mu^2 \mid \phi_\lambda, \phi_\mu, \alpha_\lambda, \alpha_\mu, l_\lambda^2, l_\mu^2, \tau_\lambda^2, \theta, \sigma^2, D\right) \\ & = f\left(\tau_\mu^2 \mid \phi_\mu, \alpha_\mu, l_\mu^2\right) \\ & \propto f_N\left(\phi_\mu \mid \alpha_\mu \cdot \mathbf{1}, K_\mu\right) \cdot f_{IG}(\tau_\mu^2 \mid a_{1\mu}, b_{1\mu}) \\ & \propto f_{IG}\left(\tau_\mu^2 \mid \frac{d}{2} + a_{1\mu}, \frac{\left(\phi_\mu - \alpha_\mu \cdot \mathbf{1}\right)^T G_\mu^{-1} \left(\phi_\mu - \alpha_\mu \cdot \mathbf{1}\right)}{2} + b_{1\mu}\right), \end{aligned} \tag{21}$$

where $G_\mu = K_\mu / \tau_\mu^2$.

- Update τ_μ^2 with a sample from

$$IG\left(\frac{d}{2} + a_{1\mu}, \frac{(\phi_\mu - \alpha_\mu \cdot \mathbf{1})^T G_\mu^{-1} (\phi_\mu - \alpha_\mu \cdot \mathbf{1})}{2} + b_{1\mu}\right). \quad (22)$$

- $l_\lambda^2 \mid \phi_\lambda, \phi_\mu, \alpha_\lambda, \alpha_\mu, l_\mu^2, \tau_\lambda^2, \tau_\mu^2, \theta, \sigma^2, D$

$$\begin{aligned} &f\left(l_\lambda^2 \mid \phi_\lambda, \phi_\mu, \alpha_\lambda, \alpha_\mu, l_\mu^2, \tau_\lambda^2, \tau_\mu^2, \theta, \sigma^2, D\right) \\ &= f\left(l_\lambda^2 \mid \phi_\lambda, \alpha_\lambda, \tau_\lambda^2\right) \\ &\propto f_N(\phi_\lambda \mid \alpha_\lambda \cdot \mathbf{1}, K_\lambda) \cdot f_G(a_{2\lambda}, b_{2\lambda}), \end{aligned} \quad (23)$$

where f_G is the density function of Gamma distribution.

- Sample $l_{\lambda 1}^2$ from $N\left(l_{\lambda 0}^2, \delta_\lambda^2\right)$, where $l_{\lambda 0}^2$ is l_λ^2 from previous iteration.
- If $l_{\lambda 1}^2 < 0$, stay unchanged.
- Otherwise, accept $l_{\lambda 1}^2$ with probability

$$r = \min\left\{1, \frac{f\left(l_{\lambda 1}^2 \mid \phi_\lambda, \alpha_\lambda, \tau_\lambda^2\right)}{f\left(l_{\lambda 0}^2 \mid \phi_\lambda, \alpha_\lambda, \tau_\lambda^2\right)}\right\}. \quad (24)$$

- $l_\mu^2 \mid \phi_\lambda, \phi_\mu, \alpha_\lambda, \alpha_\mu, l_\lambda^2, \tau_\lambda^2, \tau_\mu^2, \theta, \sigma^2, D$

$$\begin{aligned} &f\left(l_\lambda^2 \mid \phi_\lambda, \phi_\mu, \alpha_\lambda, \alpha_\mu, l_\lambda^2, \tau_\lambda^2, \tau_\mu^2, \theta, \sigma^2, D\right) \\ &= f\left(l_\mu^2 \mid \phi_\mu, \alpha_\mu, \tau_\mu^2\right) \\ &\propto f_N\left(\phi_\mu \mid \alpha_\mu \cdot \mathbf{1}, K_\mu\right) \cdot f_G(a_{2\mu}, b_{2\mu}). \end{aligned} \quad (25)$$

- Sample $l_{\mu 1}^2$ from $N\left(l_{\mu 0}^2, \delta_\mu^2\right)$, where $l_{\mu 0}^2$ is l_μ^2 from previous iteration.
- If $l_{\mu 1}^2 < 0$, stay unchanged.
- Otherwise, accept $l_{\mu 1}^2$ with probability

$$r = \min\left\{1, \frac{f\left(l_{\mu 1}^2 \mid \phi_\mu, \alpha_\mu, \tau_\mu^2\right)}{f\left(l_{\mu 0}^2 \mid \phi_\mu, \alpha_\mu, \tau_\mu^2\right)}\right\}. \quad (26)$$

We adopt an adaptive strategy to propose efficient jumping rules ($\Sigma_\lambda, \Sigma_\mu, \nu^2, \delta_\lambda^2$ and δ_μ^2). In the first phase, we adjust them to be proportional to the covariance matrices estimated from previous iterations. In the second phase, we increase or decrease the scales of these jumping distributions if the acceptance rates are too high or too low. We treat samples from the first two phases as burn-ins and only keep those from the third phase, where $\Sigma_\lambda, \Sigma_\mu, \nu^2, \delta_\lambda^2$ and δ_μ^2 are fixed, to maintain detailed balance of the chain.

Analysis of NCI-DREAM drug sensitivity data

We analyzed data from the NCI-DREAM drug sensitivity prediction challenge to demonstrate the utility of the proposed method. This data set contains dose-response measurements of 28 compounds on 53 breast cancer cell lines. Briefly, cells were treated with 9 doses of each compound in triplicate and cell counts at 72h post treatment was measured using the Cell Titer Glo assay. In the original work, the authors fit a Gompertz curve for each experiment (a cell line treated by a compound) and calculated GI_{50} (a point estimation) to quantify the sensitivity of the cell line to the compound.

We first repeated this conventional approach by fitting a Gompertz curve and a logistic curve for each data set using least square regression. We found that to find a global minimum for the mean square error, though seems straightforward, often turned out nontrivial (especially when the data sets do not follow a sigmoid pattern), in which case we tried multiple starting points manually and selected the "best" fit. This may suggest that it is difficult to "automatically" apply the sigmoid curve fitting approach to large-scale studies.

We then ran bdChemo on all data sets. For prior parameters, we set $sa_{\lambda} = 0.5, sa_{\mu} = 1, a_{1\lambda} = a_{1\mu} = 10, b_{1\lambda} = b_{1\mu} = 1, a_{2\lambda} = a_{2\mu} = 25$ and $b_{2\lambda} = b_{2\mu} = 6$. These values are chosen to control the smoothness of the fitted curve to avoid overfitting. In our software, we set them as user-specified arguments. We ran the chain for 3×10^{7} iterations and burn-in the first 2×10^{7}. We thinned the remaining sequence by keeping every 10000th draw.

We calculated the posterior mean as well as the 95% equal quantile credible intervals (CI) of $m(z), \lambda(z), \mu(z)$, GI_{50}, TGI and LC_{50} (Supplementary Table S1). In Figure S1, we compared the GI_{50}'s estimated using bdChemo and conventional Gompertz and logistic curve fitting approaches as well as grofit. Overall, the four approaches led to similar point estimations.

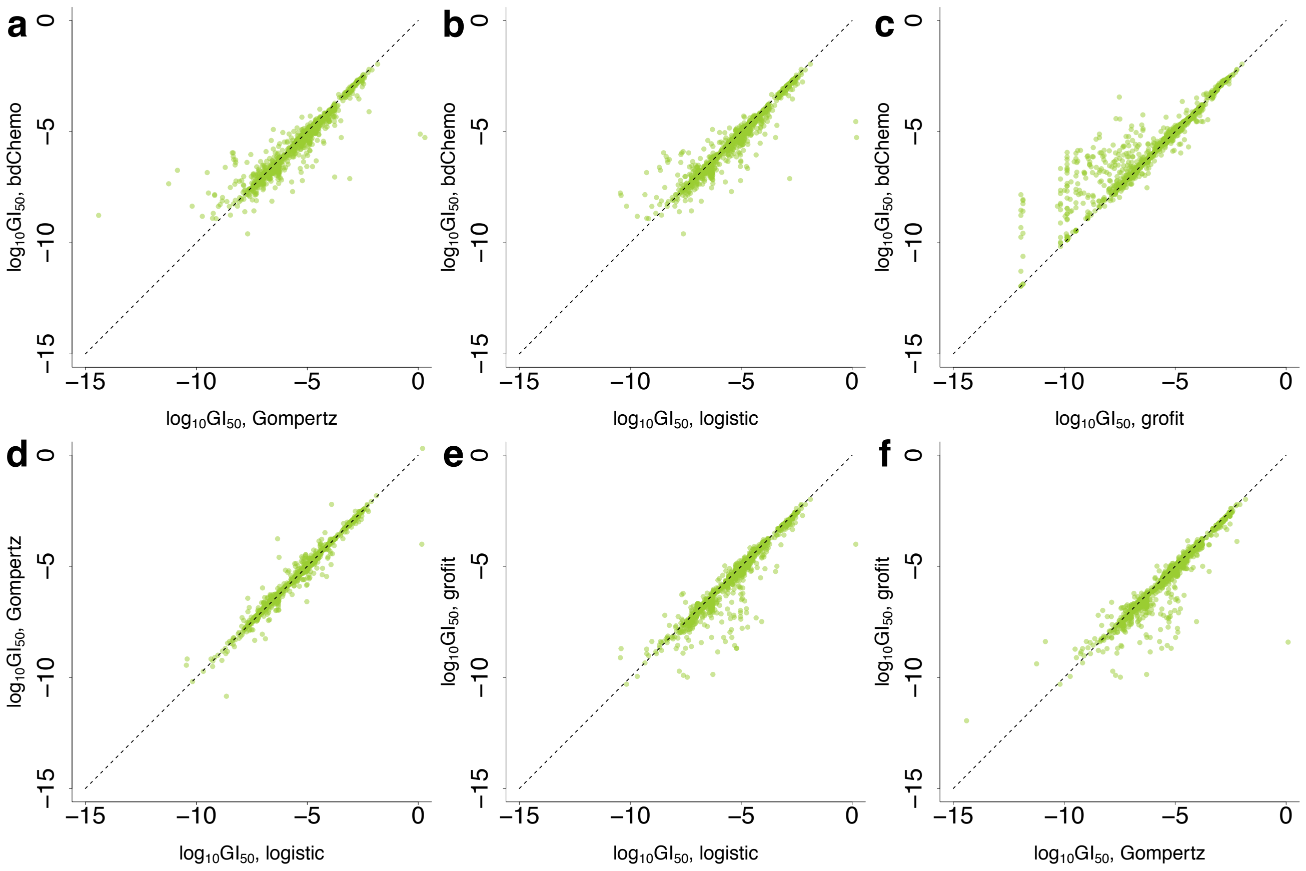

Figure S1. Comparisons of GI_{50} estimations using different approaches. (**a**) bdChemo v.s. Gompertz curve fitting; (**b**) bdChemo v.s. logistic curve fitting; (**c**) bdChemo v.s. grofit; (d) logistic curve fitting v.s. Gompertz curve fitting; (e) logistic curve fitting v.s. grofit; (f) Gompertz curve fitting v.s. grofit.

In Figure S2, we plot the birth and death rate curves, as well as their percentage changes across cell lines for each compound. We noticed that some compounds have consistent patterns across cell lines. For example, compounds such as Mebendazole, TCS PIM-11, QNZ and MG-132 demonstrate both birth inhibition and death induction effects on most cell lines. Other compounds like 4-HC, Doxorubicin, Olomoucine II, Valproate, Imantinib, Baicalein, Methylglyoxal and IKK 16 increase cell death rates on most cell lines, but their effects on cell birth rates differ by cell lines.

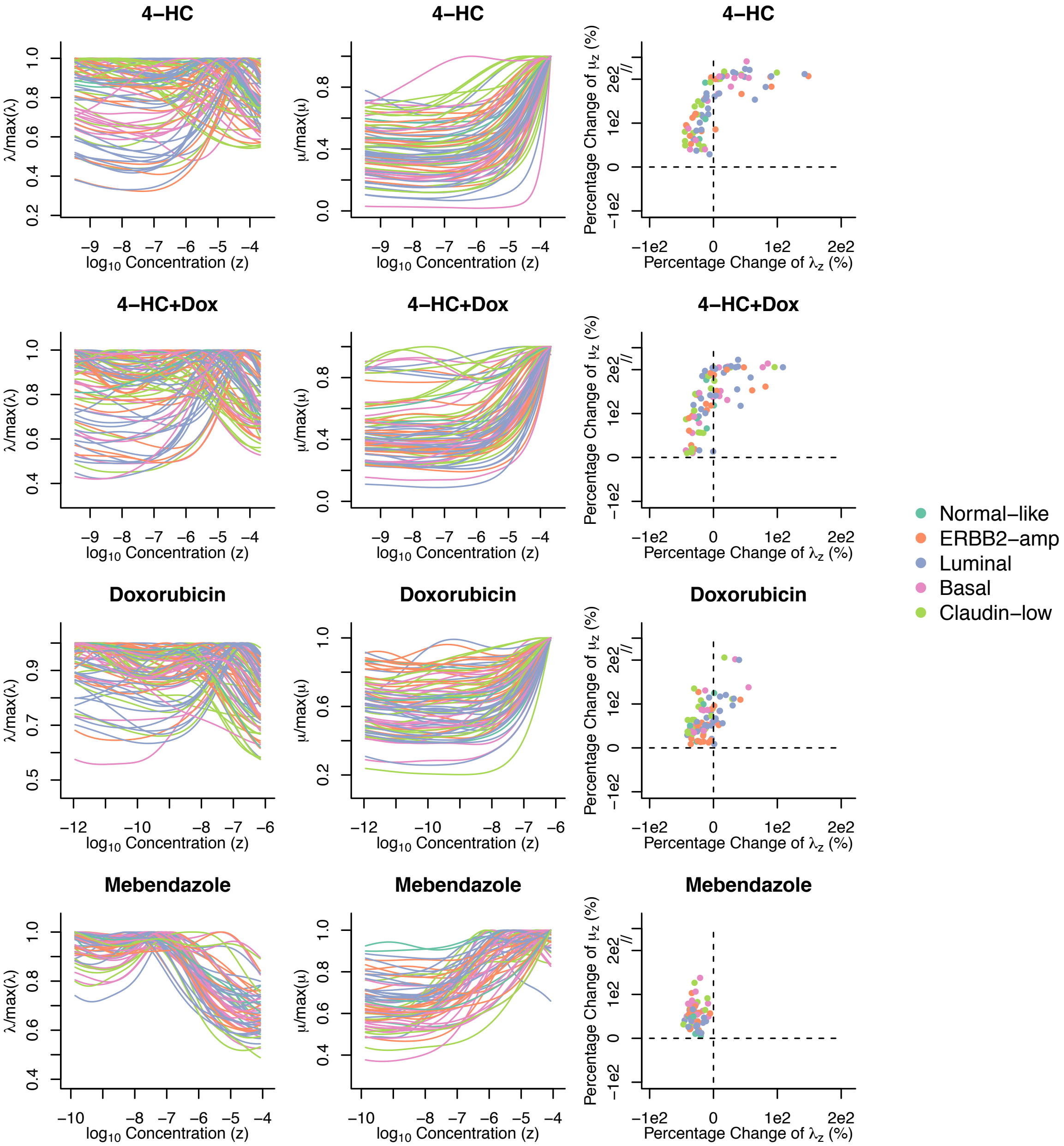

4–HC
4–HC+Dox
Doxorubicin
Mebendazole
λ/max(λ)
μ/max(μ)
log10 Concentration (z)
Percentage Change of μz (%)
Percentage Change of λz (%)
Normal–like
ERBB2–amp
Luminal
Basal
Claudin–low

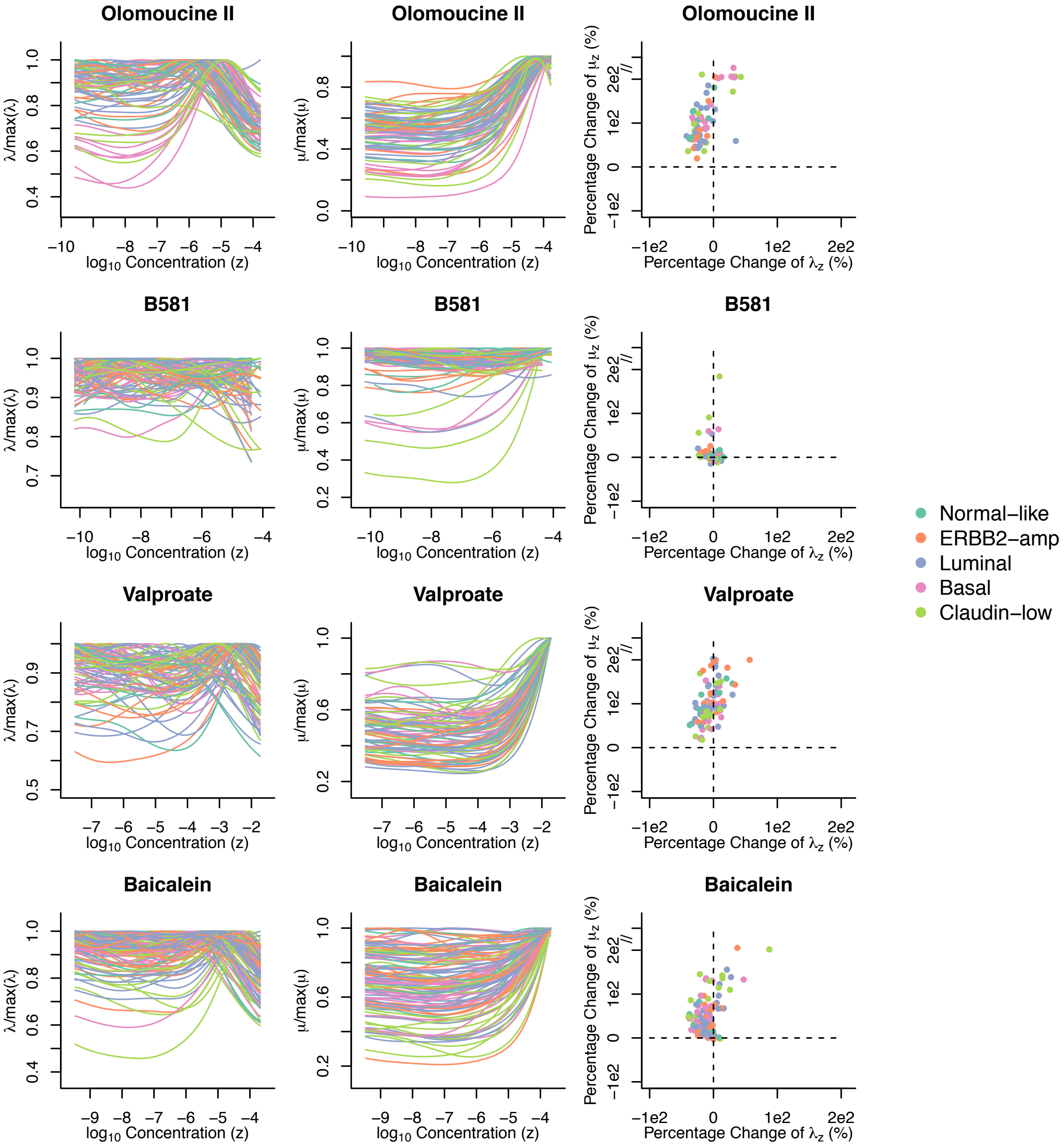

Olomoucine II
B581
Valproate
Baicalein
λ/max(λ)
μ/max(μ)
log10 Concentration (z)
Percentage Change of μz (%)
Percentage Change of λz (%)
Normal−like
ERBB2−amp
Luminal
Basal
Claudin−low

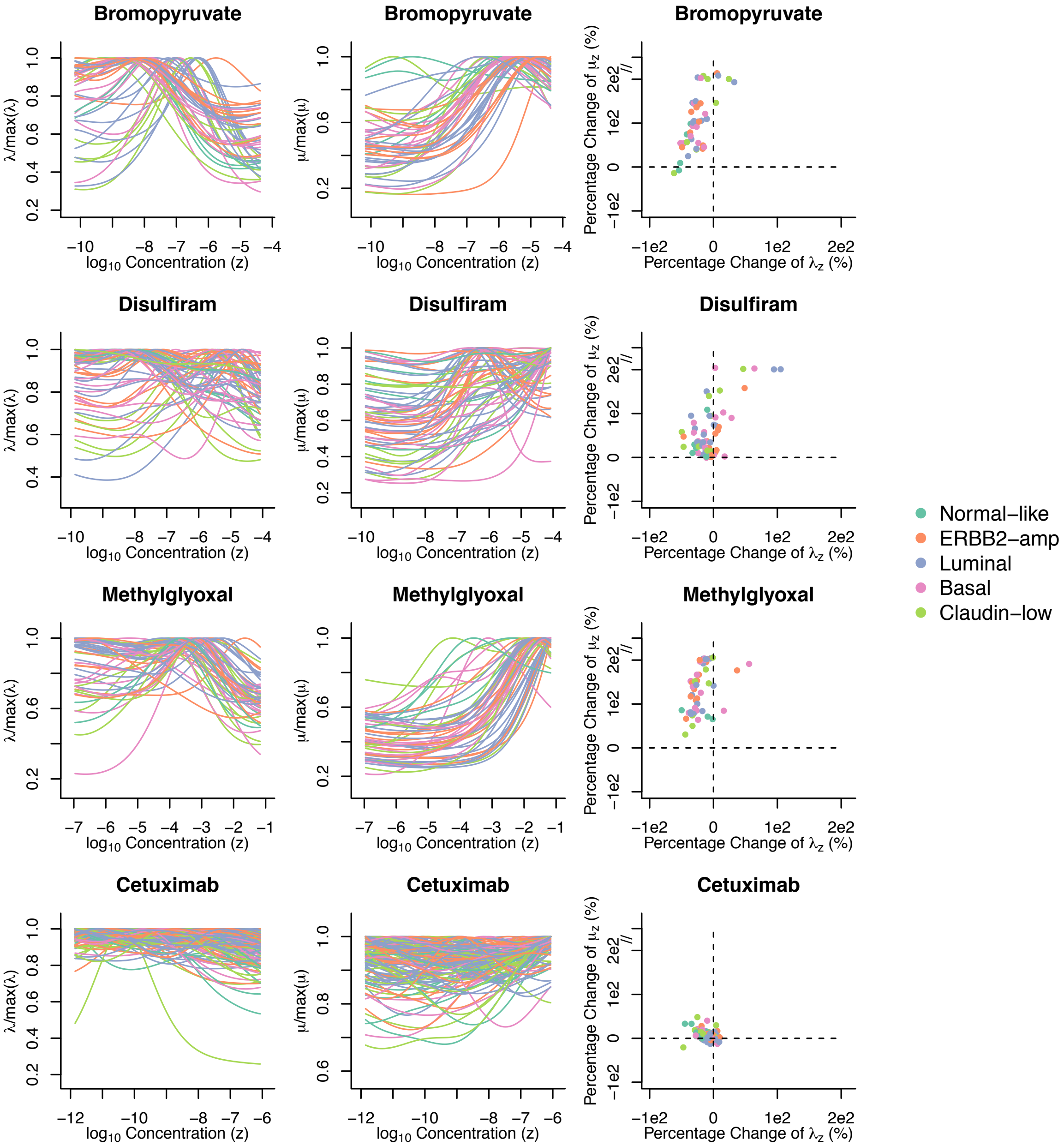
Bromopyruvate
Bromopyruvate
Bromopyruvate
Disulfiram
Disulfiram
Disulfiram
Methylglyoxal
Methylglyoxal
Methylglyoxal
Cetuximab
Cetuximab
Cetuximab
λ/max(λ)
μ/max(μ)
Percentage Change of μ_z (%)
$\log_{10}$ Concentration (z)
Percentage Change of λ_z (%)
−1e2
0
1e2
2e2
Normal−like
ERBB2−amp
Luminal
Basal
Claudin−low

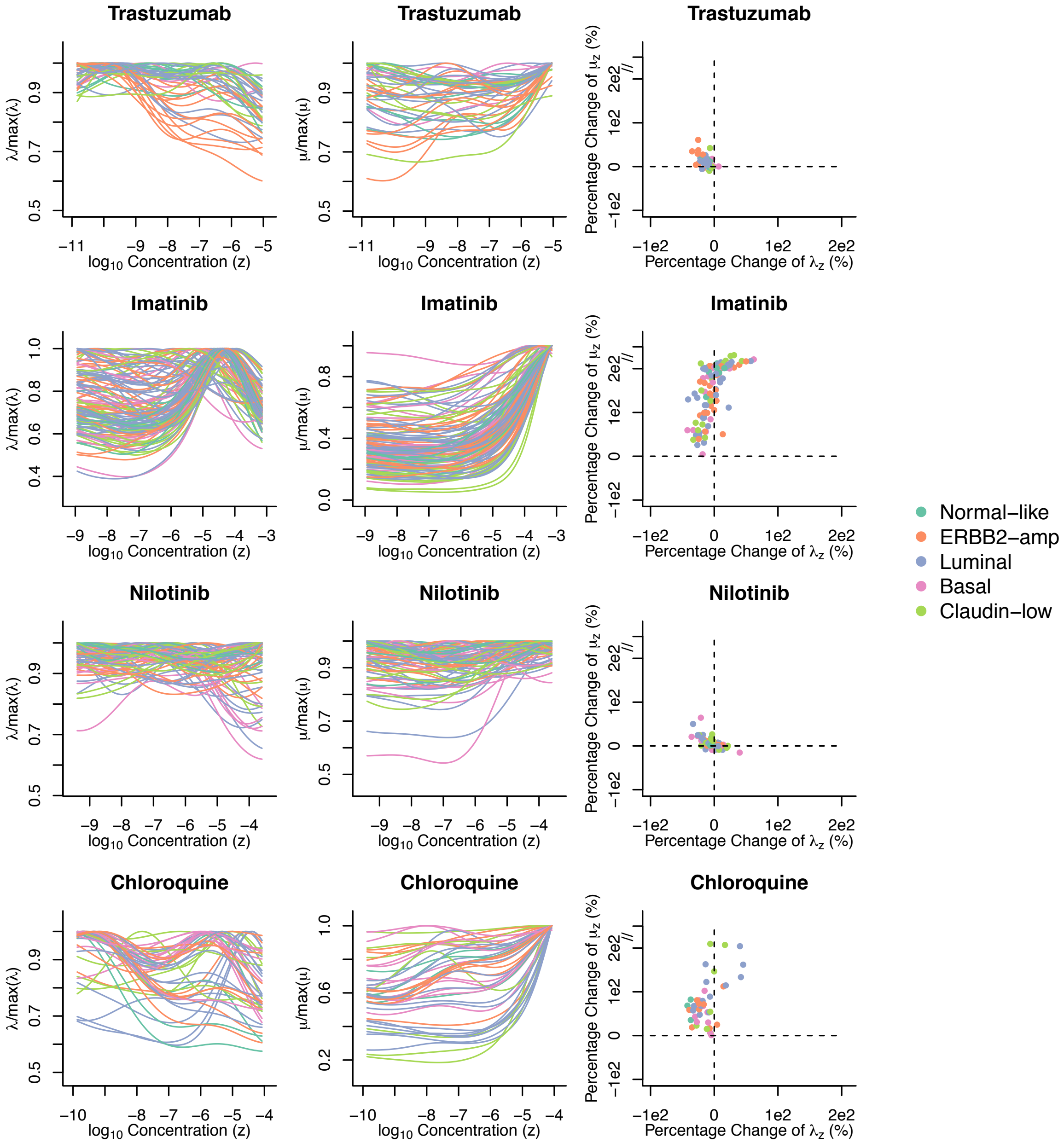

Trastuzumab
Imatinib
Nilotinib
Chloroquine
λ/max(λ)
μ/max(μ)
log10 Concentration (z)
Percentage Change of μz (%)
Percentage Change of λz (%)
Normal−like
ERBB2−amp
Luminal
Basal
Claudin−low

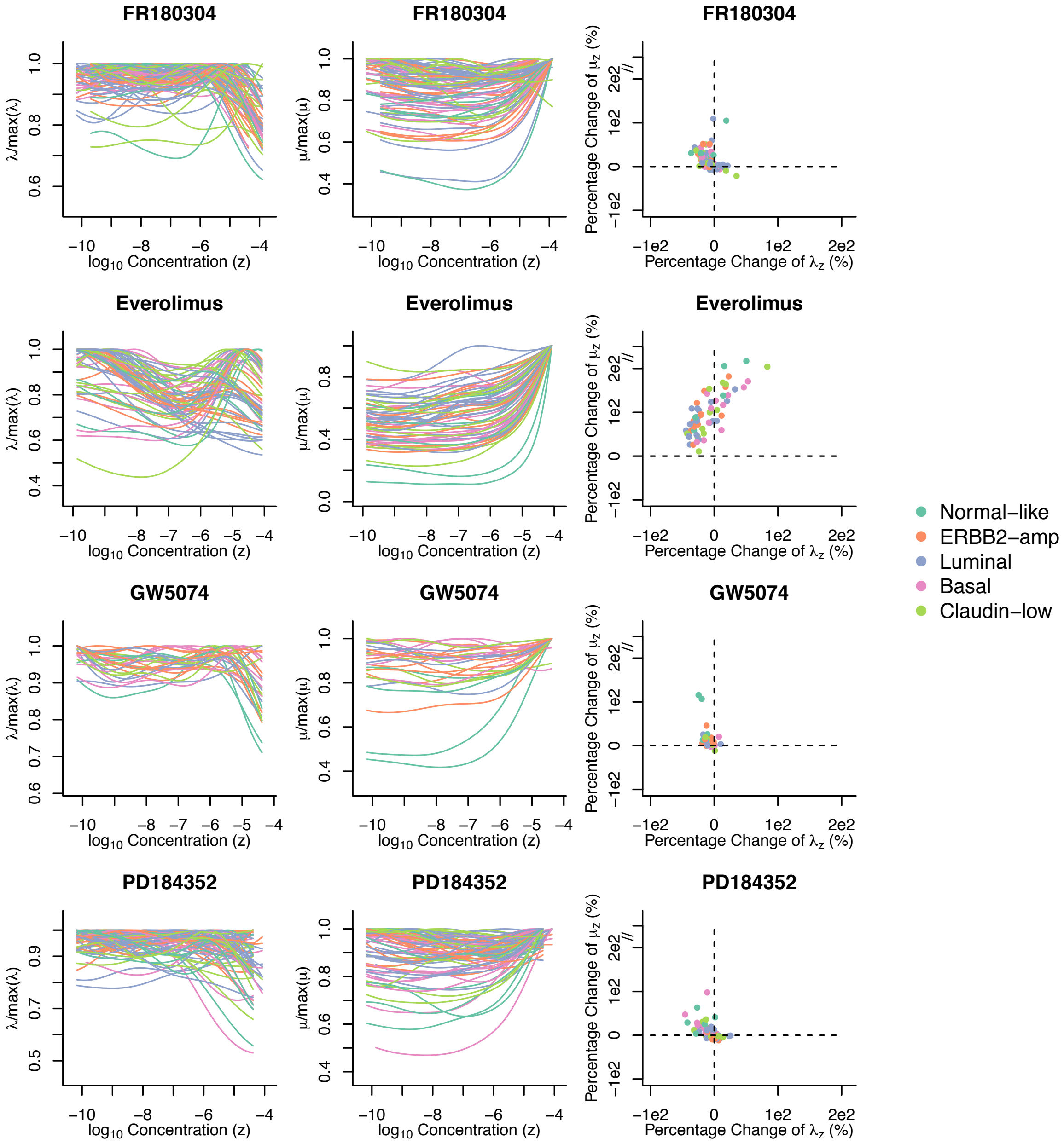
FR180304
Everolimus
GW5074
PD184352
λ/max(λ)
μ/max(μ)
log10 Concentration (z)
Percentage Change of μz (%)
Percentage Change of λz (%)
Normal−like
ERBB2−amp
Luminal
Basal
Claudin−low

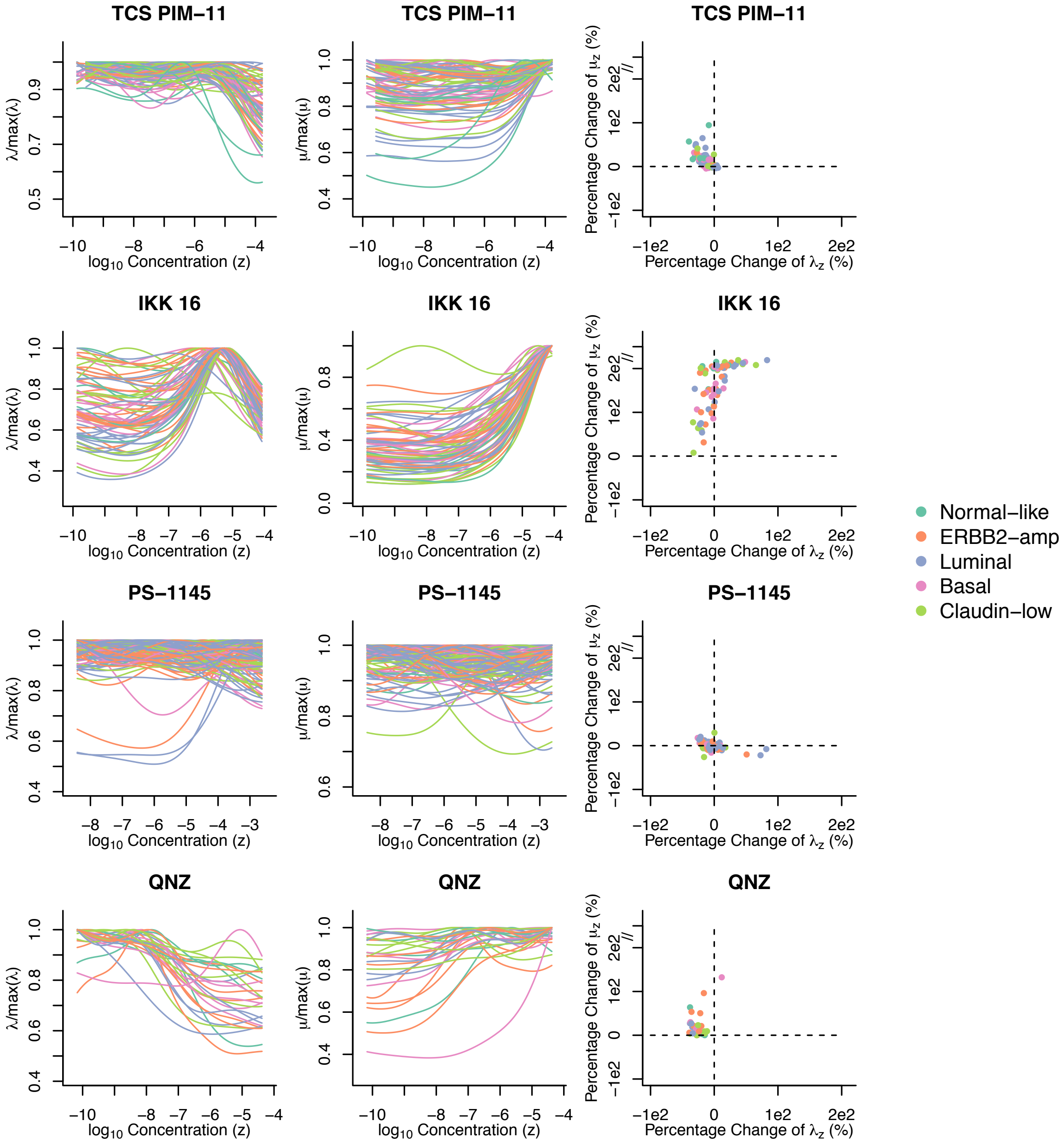

TCS PIM−11
IKK 16
PS−1145
QNZ
λ/max(λ)
μ/max(μ)
log10 Concentration (z)
Percentage Change of μz (%)
Percentage Change of λz (%)
Normal−like
ERBB2−amp
Luminal
Basal
Claudin−low

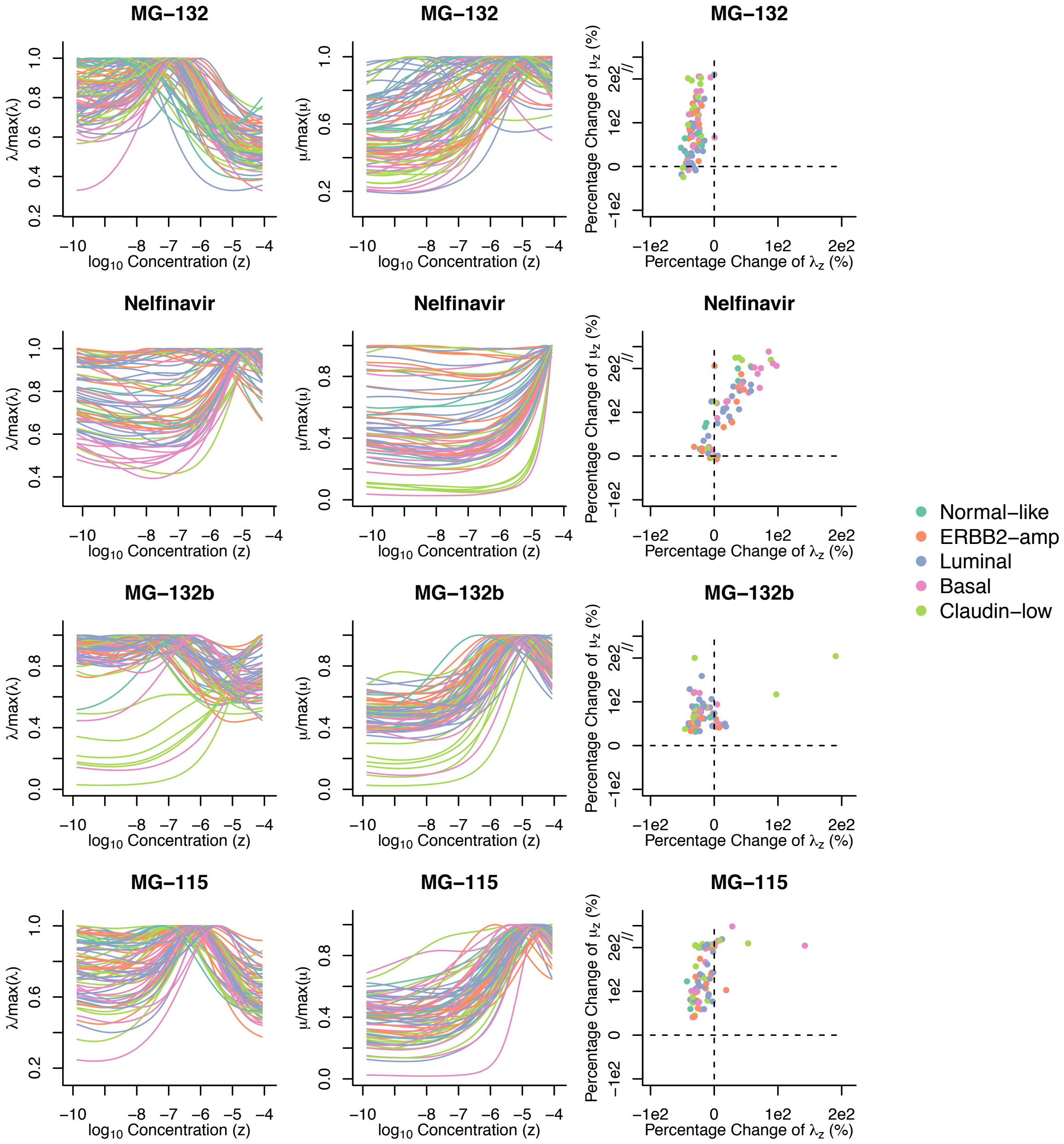

Figure S2. Birth rate (left), death rate (middle) and percentage changes of birth and death rates (right) on all cell lines for each compound. Cell lines are colored according to their subtypes[1]. Note, for illustration purpose, we normalized λ and μ curves such that they all have maximum value 1.

In Table S1 (provided as a separate Excel file), we reported the GI_{50}, TGI and LC_{50} estimations by all four methods (point estimations for Gompertz, logistic and grofit; posterior mean and 95% credible intervals for bdChemo).

In Table S2, we list the 5%, 25%, 50%, 75% and 95% quantiles of λ estimated at the lowest concentration across experiments. We can see that λ estimates are smaller than 3 on more than 75% of the datasets.

Table S2. Quantiles of λ estimated at the lowest concentrations across all experiments.

5%	25%	50%	75%	95%
0.60	1.05	1.63	2.92	7.91

In Table S3, we list the standard deviation of λ estimations across compounds for each cell line after removing outliers based on Tukey's fences criteria (11% of all datasets are removed). We observe small standard deviations here (on average 1.03; with unit births per cell per experimental time), which indicate minimum variation on the division rate for the same cell line under low compound concentration, as expected.

Table S3. Standard deviation of λ estimated at the lowest concentrations for each of 53 cell lines (11% experiments are removed as outlier using Tukey's fences criterion).

**Compound**	**sd**	**Compound**	**sd**	**Compound**	**sd**	**Compound**	**sd**
184A1	0.60	HCC1419	0.58	MCF10F	0.99	SUM159PT	1.22
184B5	0.65	HCC1428	1.01	MCF12A	1.36	SUM185PE	0.54
21MT1	1.16	HCC1569	0.95	MCF7	1.09	SUM225CWN	0.40
21NT	0.75	HCC1806	1.46	MDAMB134VI	1.66	SUM229PE	1.75
600MPE	0.88	HCC1937	1.08	MDAMB157	1.22	SUM52PE	0.54
AU565	1.95	HCC1954	1.04	MDAMB175VII	0.83	T47D	0.71
BT20	0.66	HCC202	2.50	MDAMB231	1.04	T47DKBLUC	2.23
BT474	0.47	HCC2185	0.37	MDAMB361	0.55	UACC812	0.52
BT483	0.36	HCC3153	1.13	MDAMB415	0.46	ZR751	0.43
BT549	1.06	HCC38	1.10	MDAMB453	0.62	ZR7530	0.50
CAMA1	0.62	HCC70	2.21	MX1	1.91	ZR75B	0.76
HCC1143	0.66	HS578T	1.86	SKBR3	1.42		
HCC1187	1.10	LY2	1.24	SUM1315MO2	1.01		
HCC1395	0.47	MCF10A	1.37	SUM149PT	1.70		

Reference:

1. Daemen, A. et al. Modeling precision treatment of breast cancer. *Genome Biol* **14**, R110 (2013).
2. Keiding, N. Maximum likelihood estimation in the birth-and-death process. *The Annals of Statistics*, 363-372, 0090-5364 (1975).
